# Overview of Ultrasound Detection Technologies for Photoacoustic Imaging

**DOI:** 10.3390/mi11070692

**Published:** 2020-07-17

**Authors:** Rayyan Manwar, Karl Kratkiewicz, Kamran Avanaki

**Affiliations:** 1 Richard and Loan Hill Department of Bioengineering, University of Illinois at Chicago, Chicago, IL 60607, USA; r.manwar@wayne.edu; 2 Department of Biomedical Engineering, Wayne State University, Detroit, MI 48201, USA; karl.kratkiewicz@wayne.edu; 3 Department of Dermatology, University of Illinois at Chicago, Chicago, IL 60607, USA

**Keywords:** ultrasound transducer, photoacoustic imaging, piezoelectric, micromachined, CMUT, PMUT, optical ultrasound detection

## Abstract

Ultrasound detection is one of the major components of photoacoustic imaging systems. Advancement in ultrasound transducer technology has a significant impact on the translation of photoacoustic imaging to the clinic. Here, we present an overview on various ultrasound transducer technologies including conventional piezoelectric and micromachined transducers, as well as optical ultrasound detection technology. We explain the core components of each technology, their working principle, and describe their manufacturing process. We then quantitatively compare their performance when they are used in the receive mode of a photoacoustic imaging system.

## 1. Introduction

Ultrasound transducers are devices that convert ultrasound pressure waves into electrical signal. In an ultrasound imaging machine the transducer is a transceiver device: the waves propagated from an ultrasound transducer are backscattered/reflected from an impedance mismatch in the tissue and received by the same transducer; the strength of the received pressure waves is in the range of 0.1~4 MPa [1]. Another modality that directly benefits from ultrasound transducer technology is photoacoustic imaging (PAI). PAI is an emerging modality that uses a combination of optical excitation and acoustic detection for visualizing vascular, functional, and molecular changes within living tissue [2,3,4,5,6,7,8,9,10,11]. As opposed to the optical imaging modalities such as optical coherence tomography [12] that employs ballistic photons, PAI uses diffused photons providing significantly deeper penetration. In PAI, thermoelastic expansion of tissue chromophores occurs when irradiated by a nanosecond pulsed laser—resulting in emission of acoustic waves that are then detected by ultrasound transducers for image formation [9,13,14,15,16,17]; in PAI the ultrasound transducer is a receiver device. The strength of the acoustic waves generated from the chromophores is around 800 PA·mK^−1^ [4]. The strength of the generated pressure in PAI depends on the absorption coefficient of the chromophores, the light fluence, and the characteristics of the ultrasound transducer. The lower range of the generated pressure waves in PAI compared to ultrasound imaging signifies the importance of an efficient and effective ultrasound detection technology [18]. In PAI, where the optically induced ultrasound pressure is typically weak [17], the primary requirement of the detection unit is to have a high sensitivity and a large acceptance angle over a wide range of spectral bandwidth. 

Acoustic and optical detection methods are complementary technologies that together have solved many unmet industrial and clinical needs. The contactless nature and the wavelength selectivity capability to study a particular target in the tissue (e.g., enabling functional sensing) are advantages of optical sensing over acoustic sensing, and the less penetration depth of optical sensing compared to acoustic detection technology is its disadvantage; although utilizing short wavelengths (such as X-ray), deep sensing applications are possible at a cost of ionizing the imaging target. In photoacoustic sensing, the advantages of both technologies are utilized: wavelength selectivity and adequate penetration depth; to address different unmet needs, photoacoustic technology has been implemented for different wavelengths from X-ray to infrared (IR). Due to the widely used intermediate penetration depth achieved by IR light, higher sensitivity of IR devices, and the fast growing advancement of IR optical components, most of photoacoustic systems are implemented in this regime. Infrared photoacoustic technology has been successfully used in both preclinical (to study small animal brain [19,20,21,22], eye [23,24,25,26], and skin [27,28,29]) and clinical (to detect breast cancer [30,31,32], cervical cancer [33,34], skin melanoma [35,36], and brain tumor [37,38]) applications.

Based on the ultrasound detection mechanism, transducers can be categorized into two main categories: physical ultrasound transducers and optical ultrasound detection. The physical transducers can further be classified as: (i) conventional piezoelectric, and (ii) micromachined (capacitive or piezoelectric). 

Several review studies have been conducted on ultrasound transducer technologies [39,40,41,42,43,44,45,46]. However, these studies were primarily focused on ultrasound imaging and specific to one or two technologies. The purpose of this review is to study the effectiveness of various ultrasound transducer technologies, recognize their pros and cons, and learn about their performance when they are used in the receive mode of a photoacoustic imaging system. This review does not cover the ultrasound transducer technologies that are specifically used for industrial or intravascular applications. 

The search protocol used for this review study is as follows. A PubMed database search of “transducer” AND “ultrasound” AND “photoacoustic” AND “imaging” yielded 216 results with 196 published in the last ten years. We have narrowed down the search by “piezoelectric” (45 results) and “micromachined” (29 results). Capacitive micromachined ultrasonic transducers (CMUTs) and piezoelectric micromachined ultrasound transducers (PMUTs) have been utilized in 22 and 7 articles, respectively. Moreover, 4 articles were found relevant to photoacoustic imaging among articles on optical ultrasound detection. In this study, we have reviewed a total of 189 articles.

The organization of the manuscript is as follows. First, the general design characteristics of physical ultrasound transducers are discussed. We then investigate the physical ultrasound transducers along with a quantitative analysis of their performance. Next, we discuss optical ultrasound detection technologies and present their corresponding specifications. Finally, we summarize the pros and cons of various ultrasound detection technologies and discuss their performance in photoacoustic imaging applications.

## 2. Ultrasound Transducer Characteristics

The design parameters in an ultrasound transducer are classified into two categories: (i) geometric characteristics of layers (width, length, thickness, and specific to arrays including the number of elements, kerf, and pitch size [47]), (ii) material properties (such as coupling coefficient, elastic modulus, Poisson’s ratio, density, stress coefficient, stiffness constant, acoustic impedance, and dielectric constant) used for each section. By adjusting these parameters, a transducer with a desired sensitivity, center frequency, and bandwidth is obtained. If cost is a deciding factor, sensitivity and bandwidth of the transducer may be affected. The manufacturing cost of a transducer largely depends on the fabrication process and the number of attempts needed to obtain required specifications [9]. 

Electromechanical coupling coefficient of the material represents the coupling efficiency of the transducer. In receive mode, this coefficient can be defined as the ratio between the electrical energy induced and the mechanical energy applied to the sensing material [48]. Coupling coefficient is primarily determined based on the inherent material properties of the transducer sensing elements such as stress coefficient, stiffness constant, acoustic impedance, and dielectric constant. Stress coefficient and stiffness constant are two mechanical properties that determine the elasticity of the sensing material. A highly elastic material with thermal stability is desired to build a transducer with a wide frequency range and low mechanical loss. Acoustic impedance determines the compliance of the transducer material to the target tissue material. Acoustic waves can be transmitted efficiently through the propagating medium when there is less acoustic impedance mismatch between the transducer material and the imaging target medium. Reduced acoustic impedance mismatch improves the signal-to-noise ratio (SNR) of the signal converted from the received pressure waves. In addition to acoustic impedance, there is the electrical impedance match between the transducer material and the back-end electronics (i.e., signal routing, data acquisition unit, amplifiers). Electrical impedance also affects the power transmission efficiency and SNR of the converted signal. Such electrical match is achieved using a matching network that can be realized using a material with high dielectric constant [49,50]. Moreover, a high dielectric constant is essential to improve the coupling coefficient [51].

The receive sensitivity of an ultrasound transducer is typically represented as a ratio of the detected electrical signal amplitude (in the range of micro- to milli-volts) and applied acoustic pressure (in the range of pascal to kilopascal). In photoacoustic imaging, the sensitivity is represented by noise equivalent pressure (NEP) [52], a frequency dependant metric with a unit of Pa·Hz^−1/2^. NEP is defined as the photoacoustic pressure at the imaging target that generates a transducer output equal to the noise amplitude [53]. We used sensitivity (mV/kPa) for quantitative comparison between the physical transducers and NEP (Pa·Hz^−1/2^) for transducers that work based on optical ultrasound detection methods.

An ideal ultrasound detection device should possess the following attributes: sufficiently high electromechanical coupling coefficient, an acoustic impedance that is close to tissue impedance, a large dielectric constant, low electrical and mechanical losses, low stiffness, and high thermal stability, that all together leads to a transducer with a high sensitivity over a wide spectral bandwidth. 

## 3. Physical Ultrasound Transducer Technologies

### 3.1. Piezoelectric Transducers

**Figure 1:**
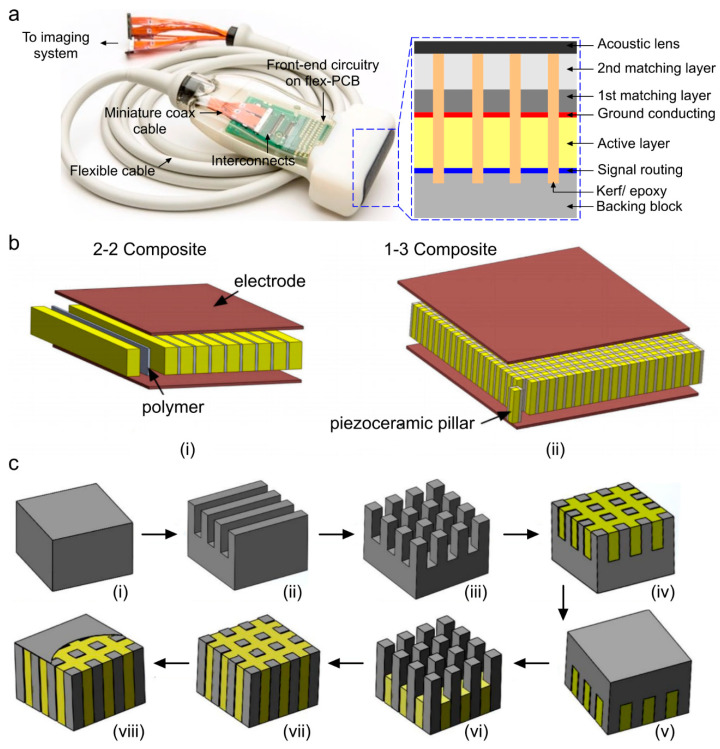
Geometric characteristics and manufacturing steps of a piezoelectric linear array ultrasound transducer. (**a**) A photograph of a piezoelectric ultrasound imaging probe; the cross-section of the sensing layer is provided in the blue dotted box [39], (**b**) structural difference between 2-2, 1-3 composite material when used in an ultrasound array transducer [62], (**c**) process flow of conventional dice and fill (DF) fabrication method using 1-3 composite and epoxy filling that includes: (i) piezoceramic material, (ii) dice in *x* direction, (iii) dice in *y* direction, (iv) epoxy filling, (v) reverse, (vi) backside dicing in *x* and *y* directions, (vii) 2nd epoxy filling, and (viii) deposit conductive layer. Reprinted with permission from [63].

Piezoelectric ultrasound transducers are the most widely manufactured and clinically available transducers that are integrated in commercial ultrasound systems [39,54,55]. The main component of a piezoelectric ultrasound transducer is piezo-material that operates based on converse and direct piezoelectric effect. In transmission mode of an ultrasound transducer, the generated acoustic waves are a result of the transient expansion and contraction of a piezo-material when exposed to an alternating electric field across the piezo-electrodes [56]. In receive mode, the incident acoustic pressure waves deform the piezo-material, and are measured in terms of the potential difference across the piezo-electrodes induced by the deformation [57,58]. A cross section of a piezoelectric linear array transducer is shown in Figure 1a. Piezoelectric ultrasound transducer elements are usually manufactured with a matching layer to reduce the impedance mismatch between the imaging target and backing layer to suppress the back scattered ringing effect. Among piezo-materials, naturally occurring crystals (quartz [59]), are seldom used in manufacturing transducers because of their weak piezoelectric performance, low dielectric and elastic properties, and low stability [54]. Engineered single crystals (such as lead magnesium niobate–lead titanate (PMN–PT) [60] and lead zinc niobate–lead titanate (PZN–PT) [61]) exhibit a high coupling coefficient and a large bandwidth that can specifically be valuable to photoacoustic imaging applications, however, the manufacturing process of these transducers is complex, expensive, and time consuming. 

The most popular piezoelectric materials are piezoceramics (such as barium titanate (BaTiO_3_) [64], lithium niobite (LiNbO_3_) [65,66], lead zirconate titanate (PZT) [67], zinc oxide (ZnO) [68]), and polymers (such as polyvinylidene difluoride (PVDF) [69]). Piezoceramics consist of randomly oriented crystallites separated by grain boundaries. They offer strong piezoelectric properties along the polarization axes, and are less expensive than polymers. Polymers such as PVDF as piezo-material alongside their copolymer trifluoroethylene (TrFE) as the thin electrode have also been found to be effective for producing high frequency transducers due to their low stiffness and improved adhesion (compliance) when compared to traditional sputtered thick metal electrodes [70,71,72,73,74]. With these polymers, low acoustic impedance (i.e., close to the tissue impedance) can be achieved at the cost of low energy conversion. To further improve the quality of piezoelectric transducers, composite materials have been developed [75]. The piezoelectric composite consists of a piezoelectric phase (piezo-ceramic) and a polymer phase (epoxy resin), with a certain connection mode, a certain volume or mass ratio, and a certain spatial geometric distribution [76]. Among different materials, PZT-epoxy resin-based composites have been the dominant material to realize the active elements of transducers in piezoelectric transducers [77,78,79,80]. Piezo-composites are classified according to respective phase connectivity (0, 1, 2, or 3) through which the phase is continuous. Since, there are two phases in piezo-composites they are referred by 2-digit numbers [76]. The first digit references the piezoelectric phase and the second digit references the polymer phase. Out of 10 conventional different combinations of connectivity [81,82], piezoelectric 1–3 [9,13] and 2–2 [14] composites are commonly used in transducer technology and are proven to exhibit high coupling coefficient with low-acoustic impedance and low stiffness, leading to improved sensitivity compared to monolithic piezo-materials [62,83]. Structural schematics of 1-3 and 2-2 piezo-composites are shown in Figure 1b. The composites are limited to low energy and low temperature applications due to their inherently low mechanical quality factor and thermal conductivity. A quantitative comparison among different types of piezo-material in terms of their determinant properties is provided in Table 1. Speed of sound (SOS) in biological tissues are in the range of 1450–1580 ms^−1^, thus it is desirable to choose a piezo-material with similar SOS. Table 1 shows that piezo-composite materials provide acoustic impedance and SOS similar to those of biological tissues, with a higher coupling coefficient as compared to polymer or ceramic based piezo-materials; that justifies the use of composites as the preferred piezo layer in ultrasound transducers.

Piezoelectric transducers can be developed as single elements or aggregated into an array (e.g., linear, convex, arc, ring, and spherical). A list of ultrasound transducer arrays that have been used in different photoacoustic imaging applications is given in Table 2. Conventionally, the arrays are realized through dice and fill method (DF) [75]. The DF method involves making a series of parallel cuts on a piece of bulk piezoelectric material with a mechanical dicing saw (Figure 1c shows the steps of a conventional DF fabrication method using 1-3 composite and epoxy filling). The material is then diced in the perpendicular direction to produce posts with a rectangular cross section. The diced material is backfilled with a polymer, then the base ceramic support is removed by lapping polishing [62,63,76]. For 2-2 composite, step 3 is skipped and the remaining steps are similar to those for 1-3 composite. Other alternative methods to make piezoelectric transducers include the interdigital bonding technique, stacked plates or lamination techniques, fiber processing, and laser machining [75]. 

One design constraint in piezoelectric transducer arrays is that the center frequency is inversely proportional to the thickness. Simultaneously, the element length (*l*) and width (*w*) to thickness (*t*) ratio of *l*:*t* ≥ 10 and *w*:*t* ≤ 0.5 must be maintained [104,105,106]. Despite the simplicity of DF method, a maximum kerf width of 10 to 15 μm can be achieved using this method and hence, manufacturing high frequency transducers (center frequency: >20 MHz) is difficult [107]. Other alternative methods such as interdigital bonding technique, stacked plates or lamination techniques, fiber processing, and laser machining, have a more complex manufacturing process and introduce non-uniformity [75]; for instance, in the laser machining approach, rapid divergence of the tightly focused laser leads to thickness non-uniformity, this inhomogeneity causes interference in the signal generated from the traducer elements. In addition to the challenges in the fabrication process, incorporation of the backing layer in piezoelectric transducers adds manufacturing difficulty to maintain layer thickness uniformity. Since medium range frequencies are commonly used for PAI of biological tissues, the DF method can be utilized to manufacture piezoelectric transducer arrays.

### 3.2. Micromachined Ultrasonic Transducers

#### 3.2.1. Capacitive Micromachined Ultrasonic Transducer (CMUT)

**Figure 2:**
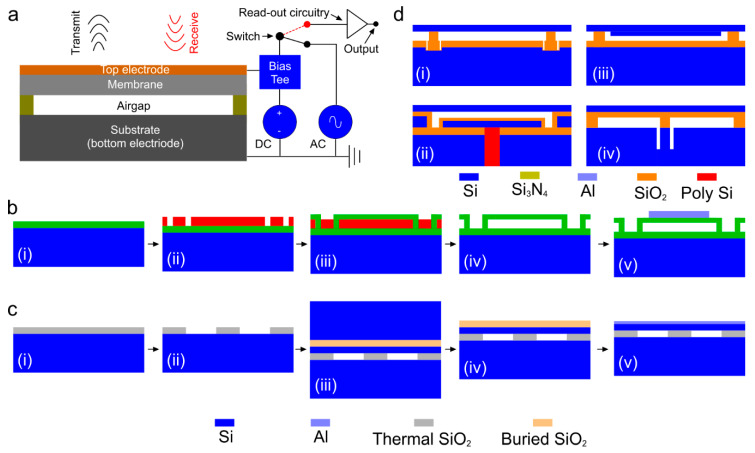
Capacitive micromachined ultrasonic transducer (CMUT) technology. (**a**) Schematic of a cross-section of a CMUT and its working principle, (**b**) steps of sacrificial release process: (i) substrate and insulation layer realization, (ii) sacrificial layer deposition and pattern, (iii) membrane layer deposition, (iv) sacrificial layer release, and (v) top electrode deposition, (**c**) steps of wafer bonding process: (i) thermal oxidation of silicon wafer (substrate), (ii) gap height and shape realization, (iii) bonding between silicon on insulator (SOI) and oxidized silicon wafer, (iv) thick silicon wafer etching, (v) buried oxide layer etching and top electrode realization [112], and (**d**) other CMUT designs fabricated using: (i) local oxidation of silicon (LOCOS) process [113], (ii) thick-buried-oxide process [114,115,116,117], (iii) mechanically coupled plate to the membrane [118], (iv) compliant post structure [114]. Reproduced with permission from [46].

Capacitive micromachined ultrasonic transducers (CMUTs) are considered to be the next generation of ultrasound transducers [108]. CMUT is an array of miniaturized capacitors consisting of suspended membranes made of silicon nitride on dielectric posts, made of silicon nitride/oxide, with a conducting layer made of aluminum/gold and a rigid silicon conducting substrate as the base with a cavity in between. Different polymer materials (e.g., bisbenzocyclobutene) have also been used as the dielectric posts and diaphragms of CMUT arrays [109,110]. As opposed to the conventional piezoelectric transducers, CMUTs rely on electrostatic principles for ultrasound wave generation and reception when a superimposed DC bias and AC signal of desired frequency is applied [111] (see Figure 2a). 

Several process flows have been proposed by various research groups to implement CMUT arrays including, surface micromachining, fusion bonding, and adhesive bonding techniques [105]. Among those, the two most common fabrication methods of CMUT arrays are sacrificial release and wafer bonding processes. The basic process flow of the sacrificial release process is as follows (Figure 2b); initially, a sacrificial layer is deposited or grown on the carrier substrate. After membrane material deposition, the sacrificial layer is etched out with an etchant, specifically chosen for sacrificial layer material and not to etch the membrane layer material [115]. Although, sacrificial release process is relatively simple, reliable, and can be achieved at lower maximum processing temperature (250 °C) [116], non-uniform effective gap height due to the roughness in the silicon nitride layer causes deviations in device performance [117]. In addition, the diaphragm may induce substantial intrinsic stress that eventually alters the device properties. The basic process flow of the wafer bonding process is as follows (Figure 2c): initially, a highly doped silicon wafer is thermally oxidized to grow a SiO_2_ layer followed by etching the oxide layer to determine the gap height and shape of the transducer elements. Next, silicon on insulator (SOI) wafer is brought in contact with the oxidized silicon wafer for bonding process [118]. The bulk silicon form SOI is removed by mechanical grinding and the buried oxide layer is etched to expose the Si diaphragm. Finally, a conducting layer such as aluminum is deposited for electrical routing. This process offers better control over gap height and thickness of the diaphragm with less residual stress. However, the wafer bonding process is very sensitive to surface roughness and cleanness that might affect the overall yield. There are a few other less popular fabrication processes such as local oxidation of silicon (LOCOS), thick buried oxide, mechanically coupled plate, and compliant post structure based CMUT arrays. The details of these processes can be found in [46]. A graphical representation of the final device structure for each method is depicted in Figure 2d.

CMUTs have gained much popularity over the last decade because they consume lower power, provide excellent electrical and thermal stability, and have a wider fractional bandwidth [46,104,105]. Micromachining techniques have advanced to allow batch fabrication of CMUT arrays of different shapes and frequencies on the same wafer with high yield and reduced price. CMUT technology has also enabled realizing densely packed elements in 2D configurations for volumetric imaging (see [119,120,121] for more details). The limitation of CMUT is that a large DC bias near the collapse voltage is required to achieve adequate sensitivity. This increases the risk of dielectric charging, changes the DC operating point which leads to an early breakdown of the device, hence greatly limiting the biomedical applicability of CMUT [111]. Hitachi Medico, Japan, Vermon, France, Butterfly Inc., USA, Kolo Medical, USA, Philips, USA, and Fraunhofer Institute for Photonic Microsystems (IPMS) are the pioneers in developing and commercializing CMUT technology. 

As compared to conventional piezoelectric transducers, capacitive transducers may offer higher sensitivity and wider bandwidth as well as higher acceptance angle. These features are all important in photoacoustic imaging, where the spectral content of PA signals is distributed over a wide frequency range [43,122]. There is extensive literature discussing the use of CMUTs in different photoacoustic applications [43,119,120,123,124]. In Table 3, the existing CMUT probes that have been used in photoacoustic imaging applications are listed. 

#### 3.2.2. Piezoelectric Micromachined Ultrasonic Transducer (PMUT)

**Figure 3:**
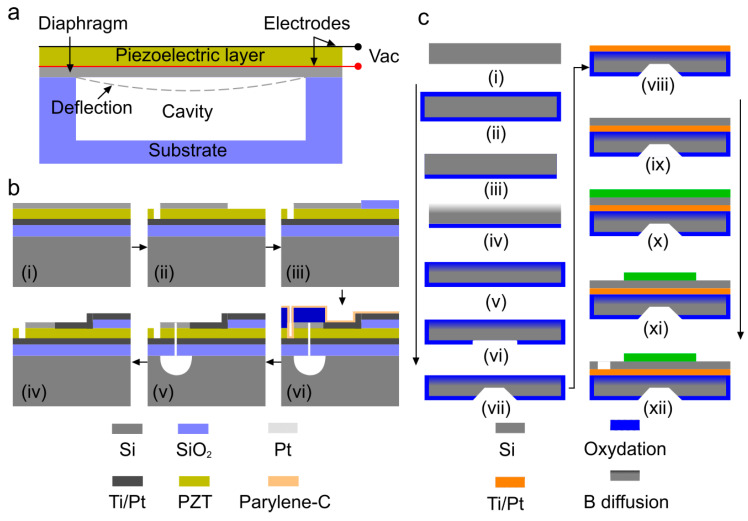
Piezoelectric micromachined ultrasound transducers (PMUT) technology. (**a**) Schematic of a cross-section of a PMUT and its working principle, (**b**) fabrication process flow of PMUTs with diaphragm defined by front-side etching method: (i) deposition of piezoelectric and electrode layer on top of oxidized silicon wafer, (i) SiO_2_, Ti, Pt, and PZT layer grown on silicon wafer, (ii) pattern top electrode, (iii) insulation pad deposition, (iv) Ti/Pt deposition, (v) etch through layers to realize bias, and (vi) release the front side diaphragm, reprinted with permission from [130], and (**c**) steps of backside etching method: (i) silicon wafer, (ii) wet oxidation, (iii) oxide etching, (iv) boron diffusion, (v) low temperature oxide growth, (vi) oxide etch, (vii) Si etch, (viii) Ti-Pt deposition, (ix) PZT deposition, (x) TiW-Au deposition, (xi) top electrode etch, and (xii) PZT etch. Reprinted with permission from [132].

Piezoelectric micromachined ultrasound transducers (PMUTs) are low-cost technology with a high sensitivity that follows the principle of piezoelectric effect. In PMUT, an ultrasound wave is generated and detected based on flexural vibration of a diaphragm similar to a thin film on a silicon substrate without any vacuum gap [40] (see Figure 3a). Apart from the classification of sacrificial layer release and reverse wafer bonding methods that are similar to those used in manufacturing CMUT (see Figure 2b,c), there are two other methods to realize PMUT array diaphragms through back and front side etching (see Figure 3b,c) [130].

The manufacturing process of PMUT with circular diaphragms released from the front-side is described in [131]. For front-side etching (depicted in Figure 3b), a silicon wafer with a platinized thermal oxide layer is used as the substrate for the deposition of the piezoelectric and electrode layer. Lithography and reactive ion etch processes are used to pattern the top electrode, etch the thin film PZT layer to expose the bottom electrode followed by the deposition of insulation layer and electrode track fan-out to bonding pads. Then, the SiO_2_/Ti/Pt/PZT/Pt thin film membranes are released from the Si substrate with XeF_2_. Finally, the devices are laminated with a 15 µm thick dry film resist to seal the etched chambers and protect the thin film stack. In backside etching [132] (depicted in Figure 3c), the fabrication process flow starts with a Si (100) wafer [29]. As is the case for surface micromachining, the process begins with preparation of the insulator, (e.g., SiO_2_ or Si_3_N_4_) on the silicon. This is then etched from one side of the Si in preparation for boron (B) doping. Boron diffusion occurs at a specific rate, allowing the control of the junction depth. After doping, the surface is cleaned and coated with low temperature oxide (LTO). Subsequently, standard photolithography is used to pattern the backside etch window. Later, the wafer is etched with an etchant such as ethylenediamine-pyrocatechol-water-pyrazine (EDP). After the back-side etching, a Ti/Pt bottom electrode is deposited by e-beam evaporation, followed by deposition of PZT and the top electrode. Finally, the top electrode and PZT are etched separately to pattern the top electrode and access the bottom electrode.

Since ultrasound transducers become smaller with increasing frequency, the effects of surface damage introduced during composite machining should be taken into account because the damaged layer volume increases in relation to the size of active piezoelectric materials. The use of a micromachining technique resolves the miniaturization issue of conventional piezoelectric transducers by realizing narrow channels or kerfs less than 10 microns, enabling high aspect ratio of piezoelectric elements [62]; this problem has been resolved in PMUT. Since the sensitivity is not limited unlike CMUTs, because they do not have a vacuum gap between the top and bottom electrodes, there is room to improve the coupling coefficient in PMUTs. Attributes of PMUTs such as low-cost with stable operation, established fabrication process, usage of popular materials (similar to conventional piezoelectric transducers) and capability of miniaturization [40,130,131,132,133] have made PMUTs a suitable candidate for photoacoustic imaging applications. Table 4 lists the studies where PMUTs have been used for photoacoustic imaging.

#### 3.2.3. ASIC Technology in Physical Ultrasound Transducers

In clinical transducer arrays, each element is connected through a long wire to the analog-front-end (AFE) unit which includes transmit and receive beamformer, preamplifier, switches, and analog-digital converters (ADCs). Although this keeps all the electronics in one place, this arrangement causes interferences and reflections along the cable [137,138]. The number of cable connections can be reduced by multiplexing, however that has negative consequences such as limited bandwidth and slower processing [139]. CMOS technology-based application specific integrated circuits (ASIC) [140] is a novel technology, applicable to micromachined transducers, that is capable of integrating the AFE along with preamplifiers immediately after the ultrasound waves are received [141]. Philips, GE, and Siemens have successfully implemented ASICs within their probes (Philips X7-2t [142], GE 6VT-D [143], Siemens Z6M [144]). ASICs are also applicable to CMUTs and PMUTs [123,134]. Recently, Kolo Medical [145] and Butterfly Network [123] have launched commercial CMUT arrays based on SiliconWave™ and CMOS technologies, respectively. Since ASICs are custom designed, they are expensive, and their repair processes are still highly complicated. 

### 3.3. Comparison between Physical Ultrasound Transducer Technologies

The physical ultrasound transducer technologies including PZT, CMUT, and PMUT are compared in terms of sensitivity, bandwidth, energy conversion and some other technical specifications in Table 5. Quantitative measurements of piezoelectric and CMUT are based on 2.43 and 2.63 MHz transducers, respectively, presented in [146,147] and that of PMUT are based on a 7~9 MHz transducer presented in [134,135].

## 4. Optical Ultrasound Detection Technologies

**Figure 4:**
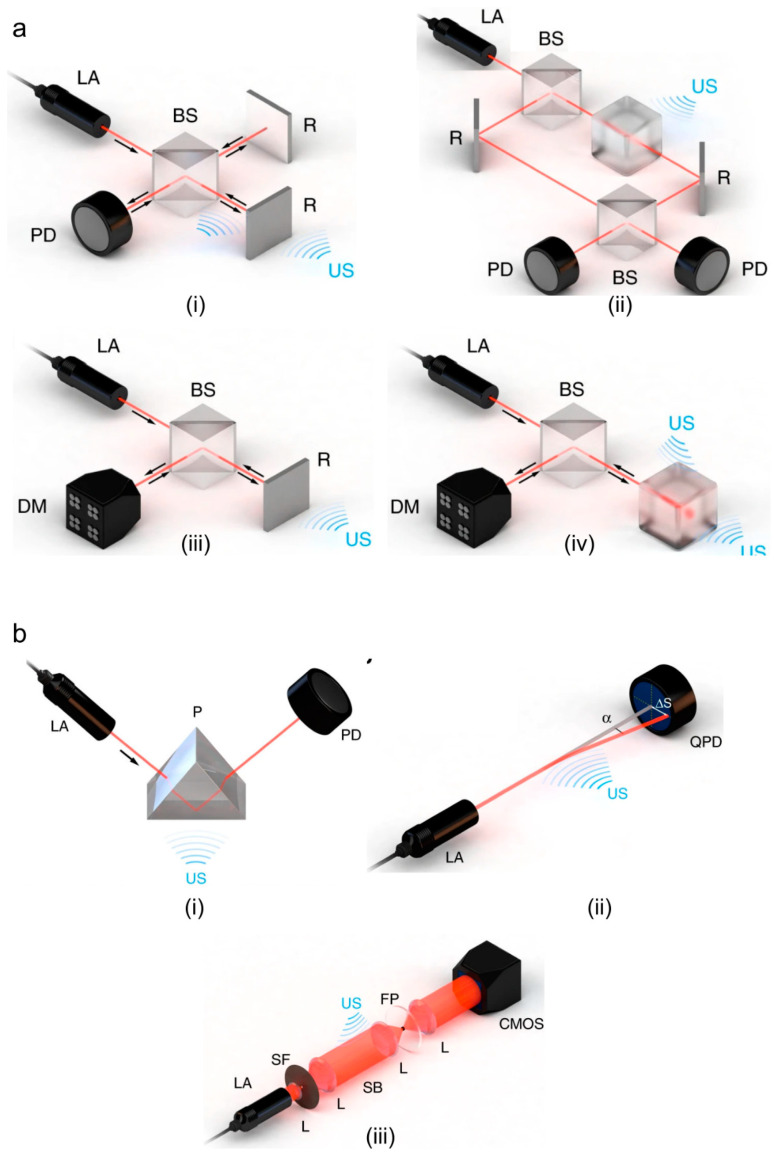
Optical ultrasound detection techniques. (**a**) Interferometric methods: (i) Michelson, (ii) Mach–Zehnder, (iii) doppler-based sensing, (iv) resonator-based sensing, and (**b**) refractometric: (i) intensity-sensitive detection of refractive index, (ii) single-beam deflectometry, (iii) phase-sensitive ultrasound detection. AL: acoustic lens, US: ultrasound, BS: beam splitter, D: detector, DM: demodulator, LA: laser, R: reflector, US: ultrasound, CMOS: complementary metal-oxide-semiconductor, FP: Fourier plane, L: lens, P: prism, PD: photodiode, QPD: quadrant photodiode, SB: Schlieren beam, SF: spatial filter. Reprinted with permission from [42].

The large size and optically opaque design of the widely used piezoelectric ultrasound transducers cause technical difficulties in some of the biomedical applications where optical illumination path and acoustic detection path must be coaxial. The mechanism that optical ultrasound detection methods offer could be a potential solution. This method employs high-finesse optical resonators to detect incident elastic waves. Providing miniaturized and optically transparent ultrasonic detectors [41], this technique yields a high sensitivity over a significantly wide frequency range, that are together ideal for photoacoustic imaging [42]. Wissmeyer et al. and Dong et al. have reviewed different methods with which optical ultrasound detection can be realized [41,42]. Based on different configurations and detection parameters, optical ultrasound detection techniques can be categorized into: (i) interferometric method and (ii) refractometric method [42]. Interferometric detection can be realized using Michelson interferometry (MI) [152,153], Mach-Zehnder interferometry (MZI) [154,155], doppler [156,157], or resonator [158,159,160]. In MI or MZI, two-beam method is employed where a laser beam passes into two optical paths, one of which is disturbed by the ultrasound wave and the other serves as a reference (see Figure 4a(i),(ii)). The changes in the optical path caused by the received pressure waves cause proportional changes in the intensity of the beam at the interferometer output [42]. In contrast to two-beam interferometers, doppler method senses ultrasound waves by measuring doppler shift (see Figure 4a(iii)). In resonator-based technique, a micron-scale optical resonator detects ultrasound waves (see Figure 4a(iv)); using this technique, miniaturization of the ultrasound detection unit is feasible. The optical resonator geometries that are most frequently used in photoacoustic imaging are Fabry–Pérot interferometers (FP) [161,162,163], micro-ring resonators (MRRs) [164,165,166], and π-phase-shifted fiber Bragg gratings (π-FBGs) [167,168,169]. Refractometric methods can be classified as intensity sensitive, beam deflectometry, and phase sensitive [41,42]. In intensity sensitive method [170,171], when the ultrasound waves pass through the interface of two media with different refractive indices, the intensity of the beam incident on that interface varies (see Figure 4b(i)). In the beam deflectometry method (see Figure 4b(ii)) [172,173], the interaction of the received acoustic waves with the medium alters the refractive index of the medium, which in turn deflects the probe beam that is eventually detected using a position-sensitive detector such as a quadrant photodiode [42]. In phase sensitive method [174], a collimated light beam passes through an acoustic field; the beam is deflected from the original path and perturbed; this beam is then focused through a spatial filter (see Figure 4b(iii)); the resultant beam is collimated and detected by a charge coupled device (CCD) or complementary metal-oxide-semiconductor (CMOS) camera; the image produced by the camera is the intensity map of the acoustic field. 

One of the major limitations of optical ultrasound detection techniques is that they are slow. Although the scanning time can be reduced by parallelization [42,175], this would increase both the complexity and cost of the detection unit [41]. Another limitation is that these configurations mostly rely on continuous-wave (CW) lasers. CW interferometry is sensitive to temperature drifts and vibrations [42]. More details about the limitations of optical ultrasound detection techniques are given in [42,176,177,178]. Performance comparison between different optical ultrasound detectors is summarized in Table 6. The sensitivity of the optical ultrasound detection methods are represented in terms of noise equivalent pressure (NEP) that is a function of frequency [42]. By multiplying the square root of the center frequency, NEP can be presented in terms of pressure unit (Pascal) as shown in [41]; we used this unit in Table 6. 

## 5. Discussion and Conclusions

During the past several years, photoacoustic imaging technology has advanced in preclinical and clinical applications [7,8,16,18,122,187,188,189,190,191,192,193]. The clinical translation of this emerging imaging technology largely depends on the future of laser technology, data acquisition systems, and ultrasound transducer technology [194]. The ideal fabrication flow of a transducer device is as follows: depending on the application requirements such as geometrical restriction, desired penetration depth, and spatial resolution, the type and technology of the transducers are determined; an optimized structural/material design is then obtained by adjusting the geometric characteristics of the transducers’ layers and their material properties; finally the transducers are built with a particular fabrication method, complexity of which depends on the budget. Ultrasound transducers with a high sensitivity in a wide spectral bandwidth, if cost is not a deciding factor, are ideal for photoacoustic imaging; a higher sensitivity can help reduce the necessary optical excitation energy and improve the penetration depth.

Among various ultrasound detection technologies, piezoelectric transducers are the most commonly used [195]; they have been made in forms of single element, as well as linear, arc, ring, hemispheric, and 2D matrix arrays. Their main limitations are that they require a matching layer, thermal instability, difficulty in realizing high frequency transducer arrays, and difficulty in miniaturization. As compared to piezoelectric transducers, CMUTs offer a higher sensitivity and a wider bandwidth, as well as a higher acceptance angle that are all important in photoacoustic imaging, where the spectral content of the PA signal is distributed over a wide frequency range [43,122]. In addition, fabrication of miniaturized transparent transducer arrays with desired shape is feasible using CMUTs. PMUT is a more recent technology with an improved bandwidth, higher sensitivity, lower acoustic impedance mismatch, flexible geometry, and the capability of CMOS/ASIC integration. PMUT, although does not outperform CMUT, relies on the established and reliable piezoelectric technology with micromachining capability. There is extensive research focused on improving the performance of PMUT that has led to promising results [40,130,133], therefore, despite better performance of CMUT, PMUT may have faster growth due to existing infrastructure. 

In comparison with physical transducers, optical ultrasound detection technologies offer higher sensitivity over a significantly wide frequency range [42]. These technologies also demonstrate the capability of miniaturization and optically transparent transducers, which are both valuable features in biomedical imaging applications where optical illumination and acoustic detection paths must be coaxial for higher efficiency; this is an ideal arrangement of illumination and detection units in a photoacoustic imaging system. The main limitations of optical transducers are high sensitivity to temperature fluctuations and vibrations, as well as system cost. Overall, NEP of the reviewed technologies, (piezoelectric: 2 mPA·Hz^−1/2^ [52], CMUT: 1.8~2.3 mPA·Hz^−1/2^ [123], PMUT: 0.84~1.3 mPA·Hz^−1/2^ [196], optical ultrasound detection: 0.45~486 mPA·Hz^−1/2^ [42]) suggest that micromachined transducers (i.e., CMUTs and PMUTs) may be the more suitable transducers for photoacoustic imaging applications. 

ASIC is a complimentary technology in transducer manufacturing to integrate the analog-front-end within the probe housing in order to reduce the noise of the transducer signal. ASIC improves the overall performance of existing transducers and therefore could help in facilitating the clinical translation of photoacoustic imaging. According to the technology market analyst projection, the usage of ASIC integrated miniature ultrasound transducer probes based on micromachined technologies will see ~18% annual compound growth rate by 2023 due to the advent of micromachining processes [197]. With the fast-growing ultrasound transducer technology, numerous computational methods have also been studied to further improve the performance of transducers by reducing noise in the transducer signal [198,199]; the result has been higher quality images [5,200,201]. With the advancement of ultrasound technology, more biomedical applications, which are currently performed using optical technologies, can be realized [12,202,203,204,205,206,207,208].

## Figures and Tables

**Table 1:** Material properties of widely used piezoelectric materials in manufacturing of ultrasound transducers [65,66,76,84,85,86].

Piezo-materials	Acoustic Impedance (MRayl)	Coupling Coefficient	Relative Permittivity	Density (kg·m^−3^)	Speed of Sound (m·s^−1^)
Quartz	13.3	0.093	4.5	2648	5000
LiNbO_3_	39	0.49	39	4700	7360
PZT	33.7	0.51	1470–1700	7500	4580
PMN-PT	37.1	0.58	680–800	8060	4610
PVDF	3.9	0.12–0.29	5–13	1780	2200
1-3 Composite	9	0.6	450	3673	1540

**Table 2:** Different configurations of piezoelectric ultrasound transducer arrays that are used in clinical applications of photoacoustic imaging. BW: bandwidth.

Application	Element no.	Configuration	Center Frequency (MHz)	BW (%)	Ref
Breast cancer	588	Hemispherical	1	130	[87]
	512	Hemispherical	2	>100	[88]
	64	Arc	1.5	130	[89]
Dermatology	Single	Spherically focused	54.2	97	[90]
			102.8	105	[91]
Vascular	Single	Focused	50	70	[92]
	256	Linear	21	66	[93]
Carotid vessel	128	Linear	5	80	[94]
	Single	Spherically focused	100	80	[95]
Musculoskeletal	32	Unfocused	6.25	80	[96]
	128	Linear	11.25	75	[97]
Adipose tissue	256	Curved	5	60	[98,99]
Thyroid	192	Linear	5.8	82.7	[100]
	64	Arc	7.5	NA	[101]
Gynecology &Urology	128	Microconvex	6.5	NA	[102,103]

NA: not available.

**Table 3:** Different configurations of CMUT that have been used in photoacoustic imaging applications.

Configuration	Element no.	CF (MHz)	BW (%)	Imaging Target	Ref
2D (16 × 16)	256	3.48	93.48	Fishing line filled with ICG, pig blood, and mixture of both	[120]
2D (16 × 16)	256	5	99	Tube filled with ink	[125]
2D (16 × 16)	256	5.5	112	Hair sample in tissue mimicking phantom	[119]
2D (Transparent)	NR	3.5	118	Wire phantom	[126]
2D (Transparent)	Single	1.46	105	Pencil lead; loop shaped tube filled with ICG	[126]
2D (Transparent)	NA	2	52.3	Characterization with hydrophone	[127]
Ring	NA	3	NA	Two polyethylene tubes	[128]
Hemisphere (spiral) *	500	4	>100	Arterioles and venules	[129]

BW: bandwidth; CF: center frequency, NA: not available. * Clinical application.

**Table 4:** Different configurations of PMUT that have been used in photoacoustic imaging applications.

Configuration	Element no.	CF (MHz)	BW (%)	Imaging Target	Ref
Linear	65	6.83	29.2	Six pencil leads at different depths	[134]
Linear	80	7	68%	Four pencil leads at different depths	[135]
1.5D Endoscopic	256 (32 × 16)	5	30	Metal spring; tricuspid valve and right ventricle in a porcine model	[136]

BW: bandwidth; CF: center frequency.

**Table 5:** Comparison between physical ultrasound transducer technologies. DF: dice and fill, IC: integrated circuit, DC: direct current.

Parameters	Piezoelectric (PZT) [146,147]	CMUT [146,147]	PMUT [134,135]
Method	DF, Laminating	Wafer bonding, micromachining	Micromachining, wafer transfer
Sensitivity (mV/kPA)	4.28	22.57	0.48
Bandwidth (%)	60–80	≥100	50–60
Energy conversion (%)	45–75 [148]	>80	2.38–3.71
SNR (dB)	18–22 [149,150]	22–87 [120,151]	10–46
IC integration	Not compatible	Compatible	Compatible
Matching layer	Required	N/A	N/A
DC bias	N/A	Required	N/A

**Table 6:** Summary of the performances of different optical ultrasound detection techniques. Reproduced from [41,42].

Method	Configuration		Readout Element Diameter/Dimension (μm)	Detection Geometry	BW (MHz)	NEP (Pa)	Ref.
Interferometric	MI		**	Point; disk	5; 20	35; 275	[179]
	MZI (free space)		90	Bar	17.5	100 (x mm)	[176]
	MZI (fiber optic)		125/8	Bar	50	92 × 10^3^ (x mm)	[180]
	Doppler		-/12	Point	10	-	[181]
	RI	FP (free space)	**	Bar	25	20	[182]
		FP (fiber optic)	125/8	Bar	50	1	[180]
		MRR (integrated)	60/0.8 × 0.8	Ring	140	6.8	[183]
		FBG (fiber optic)	125/8 × 100	Bar	20	450	[184]
		FBG (integrated)	500/1.5 × 1.5	Bar	60	6.5 × 10^3^	[185]
Refractometric	Intensity-sensitive		15 × 10^−3^	Prism	100	100 *	[186]
	Deflectometry		90	Needle beam	17	2.76 *	[172]
	Phase-sensitive		10^−2^	Schlieren	110	486 *	[174]

* Unit: mPA·Hz^−1/2^, ** Diffraction limited, BW: bandwidth; NEP: noise equivalent pressure, MI: Michelson interferometry; MZI: Mach–Zehnder interferometry; DI: doppler- interferometry; RI: resonator- interferometry.
